# Veterans’ positive parenting practices and satisfaction over time: Examining the impact of moral injury, posttraumatic stress, and internalizing symptoms

**DOI:** 10.1002/jts.70083

**Published:** 2026-06-01

**Authors:** Ryan P. Chesnut, Jennifer K. Karre, Cameron B. Richardson, Keith R. Aronson, Daniel F. Perkins

**Affiliations:** ^1^ Clearinghouse for Military Family Readiness The Pennsylvania State University University Park Pennsylvania USA; ^2^ Department of Biobehavioral Health The Pennsylvania State University University Park Pennsylvania USA; ^3^ Department of Agricultural Economics, Sociology and Education The Pennsylvania State University University Park Pennsylvania USA

## Abstract

Little is known about the association between moral injury (MI) and parenting. Although there is evidence that psychological symptoms impact veterans’ parenting, few longitudinal studies exist. The current study used parallel process latent growth curve models to explore the effects of MI and other psychological symptoms on positive parenting practices and satisfaction in a sample of U.S. veterans who served following the September 11, 2001, terrorist attacks (*n* = 1,078). On average, positive parenting practices, α = −.09, *p* < .001, and satisfaction, α = −.13, *p* < .001, declined over time. MI from betrayal and depressive symptoms negatively predicted baseline positive parenting practices, *B*_MI‐Betray_ = −0.04, *p* = .004; *B*_Depression_ = −0.08, *p* < .001, and parenting satisfaction, *B*_MI_ *_‐Betray_* = −0.04, *p* = .026; *B*_Depression_ = −0.08, *p* < .001, and MI from self‐induced (in)action negatively predicted baseline positive parenting practices, *B* = −0.04, *p* = .036. Posttraumatic stress disorder (PTSD) symptoms were negatively associated with positive parenting practices, *B* = −0.04, *p* = .010, and parenting satisfaction, *B* = −0.06, *p* = .001, over time. MI appears to have a negative, short‐term impact on parenting behaviors and satisfaction within the first 3 months of military separation. PTSD symptoms seem to exert a persistent, negative effect on parenting, which aligns with previous research in the military and veteran context.

Moral injury (MI) encompasses various negative biological, psychological, social, and spiritual symptoms (e.g., guilt, shame, loss of faith, social withdrawal) associated with violations of deeply held moral convictions (Litz et al., 2009; Litz & Walker, 2025). Though a complex phenomenon without definitional or theoretical consensus (Litz & Walker, 2025), MI is thought to arise from two separate, yet related, transgression types. In the first type, *self‐directed* transgressions, individuals may commit an act that misaligns with, or fail to act in a way that aligns with, their moral code. In the second type, *other‐directed* transgressions, an individual may witness or have been the target of someone else's action or inaction that is deemed morally reprehensible. Recently, some scholars (e.g., VanderWeele et al., 2025) have posited that MI can also emerge from one's experiences with natural phenomena or personal struggles. Regardless of the originating source, the inability to successfully resolve or cope with the distress that stems from experiences of moral violations is theorized to lead to MI (Litz et al., 2009; Litz & Walker, 2025).

Though MI is considered a distinct psychological phenomenon (Litz & Walker, 2025), it is positively associated with posttraumatic stress disorder (PTSD), anxiety, and depression (Hall et al., 2022). Among U.S. service members and veterans, the negative impact of PTSD on parenting behaviors and parenting satisfaction is well documented (Kizer & Le Menestrel, 2019). Anxiety and depression are also thought to negatively impact parenting in this population.

## Mental health and parenting

How parents actually parent is “fundamentally shaped by their mental health” (Hoghughi, 2004, p. 11). Most parenting models (e.g., attachment theory, role theory, process of parenting model) consider factors that impact a parent's ability to be emotionally available and provide children with stability, safety, and suitable limits as risks that negatively affect child well‐being, development, and adjustment (Ryan et al., 2017; Smith, 2004). Several conceptual pathways between parental mental health and parenting disruptions have been identified. These parenting disruptions include self‐preoccupation, emotional unavailability, threats of abandonment, unpredictable or unsuccessful limit‐setting, irritability and overreactions, and family discord and hostility (Reder & Duncan, 2005).

Although some parents who experience mental health challenges have little to no disruption in parenting (Smith, 2004), others exhibit parenting problems. For example, parental anxiety is related to less parental connection, more psychological control, less involvement, less authoritative parenting, and more punitive parenting (Apsley & Padilla‐Walker, 2020; Leinonen et al., 2003). Similarly, parental depression is associated with less parental connection and involvement (Apsley & Padilla‐Walker, 2020), more punitive and less authoritative parenting (Leinonen et al., 2003), more negative affect toward children, diminished sensitivity, and poor discipline practices (Berg‐Nielsen et al., 2002). Parental depression is also related to more parenting stress, worse parenting function, and less parenting satisfaction (Lovejoy et al., 2000). Moreover, reviews of the literature have found that PTSD is related to parenting stress, less effective parenting behaviors, decreased parenting alliance, lower parenting sense of competence, less parenting satisfaction, less involvement, less sensitive parenting, and harsh parenting (Christie et al., 2019). Collectively, the extant literature demonstrates that mental health exerts a considerable influence on how parents view, understand, and enact their parenting roles.

When considering mental health and parenting in military and veteran populations, the *cognitive behavioral interpersonal theory* of PTSD (C‐BIT) has been proposed as a framework for understanding how PTSD can negatively influence parenting cognitions, attitudes, and behaviors (Creech & Misca, 2017). C‐BIT proposes that cognitive, emotional, and behavioral disruptions stemming from PTSD symptoms directly impact how parents perceive, connect, and engage with their children. Though C‐BIT focuses on PTSD, the overlap in symptoms among PTSD, MI, depression, and anxiety, coupled with the way in which these constructs affect emotional and behavioral regulation (Litz & Walker, 2025), indicates that the underlying mechanisms of the C‐BIT framework could be extended to mental health outcomes beyond PTSD.

## Moral injury and parenting

Although the effects of MI on the family system remain understudied, the C‐BIT model provides theoretical justification for the impact MI—especially self‐directed and other‐directed MI symptoms—may have on parenting. When individuals behave in ways that are at odds with their moral code, this may cause internal conflict upon return to their family system. Difficulty reconciling one's behavior with their moral code may lead to emotional disturbances, which, as theorized by Creech and Misca (2017), may lead to aggression, diminished closeness, and diminished emotions. Witnessing morally injurious acts or experiencing betrayal may impact parents’ view of the world as being just. This perspective can, then, impact parents’ cognitive processes or lead to behavioral avoidance, which may, as outlined in the C‐BIT model, impact affective engagement and/or parenting behaviors.

Studies have slowly begun to investigate the connection between MI and family life (e.g., Haight et al., 2017; Koenig et al., 2018; Maguen et al., 2020; Ne'eman‐Haviv et al., 2025; Williamson et al., 2023), but the effects of self‐directed and other‐directed MI symptoms on parenting outcomes remain understudied (Haight et al., 2017; Maguen et al., 2020; Ne'eman‐Haviv et al., 2025). For example, in Maguen and colleagues’ (2020) study, self‐directed and other‐directed MI experiences were found to be related to increased impairment in positive parenting practices for male and female veterans. Ne'eman‐Haviv and colleagues’ (2025) study found that self‐directed MI (labeled MI‐Shame in the study) negatively predicted parenting satisfaction and practices and parent–child relationship quality. Furthermore, exposure to potentially morally injurious experiences (PMIEs) had a negative indirect effect on parenting through PTSD symptoms and self‐directed MI symptoms, as well as through parental beliefs about children's anxiety via PTSD and self‐directed MI symptoms. Although prior research has provided valuable insights that have advanced the field, these studies were cross‐sectional and did not control for other relevant, comorbid psychological constructs (e.g., PTSD, anxiety, depression) that are associated with parenting outcomes.

## Present study

For the present study, we sought to build on prior cross‐sectional work by examining parenting through a longitudinal lens. We hypothesized that MI, a psychological construct that impacts emotional responsivity, which is theorized to be a linking mechanism among anxiety, depression, PTSD, and suboptimal parenting (Creech & Misca, 2017), would uniquely predict decreases in positive parenting practices and satisfaction over time above and beyond the influence of anxiety, depressive, and PTSD symptoms. Parallel process latent growth curve modeling (PP‐LGCM; Preacher et al., 2008) was used to examine baseline levels and changes in self‐reports of positive parental practices and satisfaction among U.S. veterans who served in support of military operations following the September 11, 2001, terrorist attacks (9/11; see Figure 1). MI and symptoms of PTSD, depression, and anxiety were treated as time‐invariant predictors (i.e., measured at one time), and their effects were investigated simultaneously while controlling for relevant demographic and military characteristics. Specifically, based on prior theoretical and empirical studies (Gupta et al., 2023; Morgan et al., 2022), parent gender, pay grade, combat exposure, marital status, and number of deployments were included as covariates. Given the theoretical emphasis placed on the separate roles of perpetrating or failing to act, witnessing, and being betrayed in the etiology and presentation of MI (Litz et al., 2009), each MI subtype, rather than an overall MI construct, was simultaneously included in the model to estimate the effect of each subtype in the presence of the other subtypes, relevant mental health constructs, and pertinent covariates. Further, as the predictor variables were treated as time invariant, potential mediation pathways were not examined to avoid the biases that are typically associated with cross‐sectional mediation analyses (Maxwell & Cole, 2007).

**FIGURE 1:**
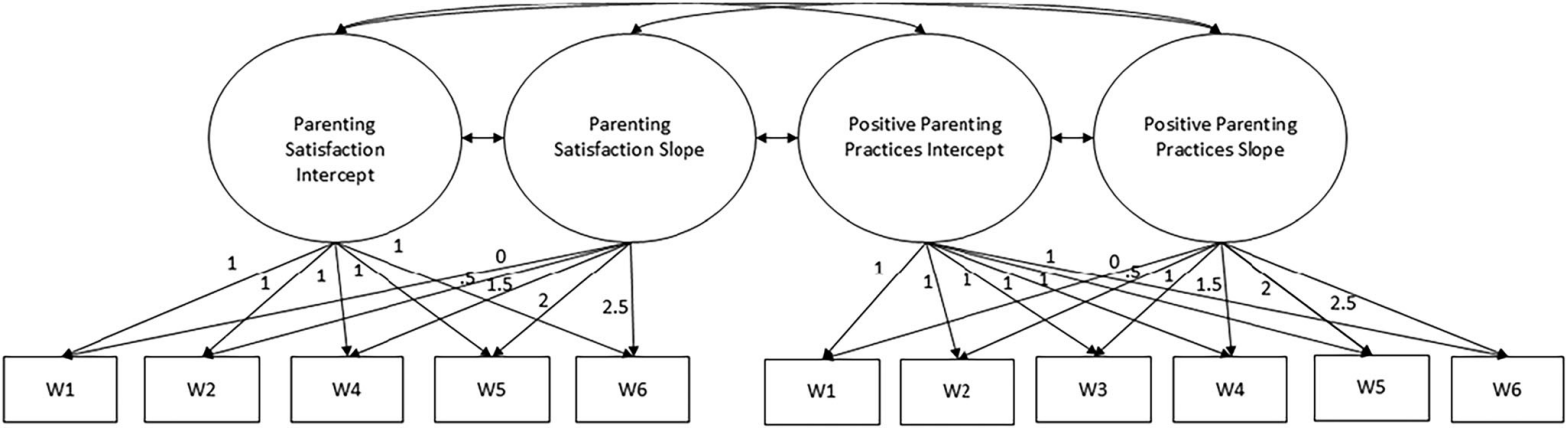
General depiction of parallel process latent growth curve modeling (PP‐LGCM) *Note*: Observed indicator residual variances and residual covariances among concurrent manifest indicators are not shown for presentational clarity. The figure depicts a linear PP‐LGCM. Nonlinear models (e.g., quadratic, latent basis) are also possible. A survey administration error led to parent satisfaction not being assessed at Wave (W) 3.

## METHOD

### Participants and procedure

Participants were a subsample of post‐9/11 veterans who participated in The Veterans Metrics Initiative (TVMI) and reported having children 0–18 years old (*n* = 1,078). TVMI was a longitudinal study that followed post‐9/11 veterans for 2.5 years (Vogt et al., 2018). The Veterans Affairs/Department of Defense Identity Repository (VADIR) was used to identify all post‐9/11 veterans who had separated from the military between August 2016 and November 2016 (*N* = 48,965). These individuals were invited to participate in a study on military‐to‐civilian transitions. Of those invited, 9,566 individuals consented to participate and completed the Wave (W) 1 survey. Surveys were readministered every 6 months for a total of six waves (W1–W6) of data collection. The parent subsample included veterans who reported having the same number of children between 0 and 18 years of age at each wave. This sampling approach was used to control for the potential influence of parental transitions (e.g., having a new baby, a child moving out of the house) that may impact parenting practices and satisfaction. The demographic and military characteristics of this parent subsample are presented in Table 1.

**TABLE 1:** Participant demographic characteristics

Characteristic	%	*M*	*SD*	Range
Gender^a^				
Male	85.3			
Female	14.7			
Race/ethnicity^b^				
White	73.4			
Black	7.7			
Hispanic	10.9			
Other racial/ethnic group	7.9			
Service branch/component				
Army	31.1			
Navy	19.1			
Air Force	23.5			
Marines	14.5			
National Guard/Reserves	11.9			
Marital status				
Never married	2.6			
1st and Only marriage	68.6			
2nd or later marriage	19.8			
Separated/divorced/widowed	9.1			
Pay grade				
E1–E4 junior enlisted	18.2			
E5–E6 mid–grade enlisted	30.6			
E7–E9 senior enlisted	22.4			
W1–W5 warrant officers	1.9			
O1–O3 junior officers	6.6			
O4–O10 senior officers	20.3			
Parent age^c^		36.41	7.78	20.30–60.80
Number of deployments		2.25	2.19	0–10
Combat exposure^d^		6.22	7.66	0–27
Number of children < 18 years old		1.94	0.93	1–6

*Note*: *N* = 1,078. Percentages may not sum to 100.0 due to rounding. All demographic characteristics were measured at Wave 1.

^a^ Gender breakdown was used to define parent type (i.e., Male = father; Female = mother).

^b^ *n* = 1,072.

^c^ *n* = 1,056.

^d^ Combat exposure was measured with the Deployment Risk and Resilience Inventory–2 Warfare Exposure–Short Form.

This study was performed in line with the principles of the Declaration of Helsinki. Ethical approval was obtained from ICF International, Inc. Informed consent was obtained from all participants included in the study. Prior to starting the TVMI Wave 1 survey, participants were presented with an informed consent form and indicated their consent to participate in the study by clicking a checkbox.

### Measures

#### Positive parenting practices

Positive parenting practices were assessed at all six waves using the five‐item Parental Functioning subscale of the Well‐Being Inventory (Vogt et al., 2019). Items assess the occurrence of constructive and supportive parenting behaviors over the past 6 months (e.g., “How often have you been actively involved in your children's activities?”) and are rated on a 5‐point Likert‐type scale ranging from 1 (*never*) to 5 (*most or all of the time*), with higher scores reflecting more use of positive parenting practices. The subscale has been shown to have satisfactory internal reliability, construct validity with respect to relevant domains of functioning and satisfaction, as well as known‐groups validity in terms of differentiating between veterans with and without a probable mental health diagnosis. A mean score (possible range: 1–5) was calculated at each wave. In the present sample, the Parental Functioning subscale demonstrated good internal consistency across study waves, Cronbach's αs = .85–.88.

#### Parenting satisfaction

Parenting satisfaction was assessed at five waves using the three‐item Parental Satisfaction subscale of the Well‐Being Inventory (Vogt et al., 2019). Due to a survey administration error, the subscale was not administered at W3. Items assess the degree of satisfaction experienced in the parenting role over the previous 6 months (e.g., “How satisfied have you been with how close you are with your child(ren)?”) and are rated on a 5‐point Likert‐type scale ranging from 1 (*very dissatisfied*) to 5 (*very satisfied*), with higher values denoting higher levels of parenting satisfaction. This subscale has been shown to have satisfactory internal consistency, construct validity in regard to pertinent domains of functioning and satisfaction, and known‐groups validity in terms of distinguishing between veterans with and without a probable mental health diagnosis. A mean score (possible range: 1–5) was calculated at each wave where data were available. In the present sample, the Parenting Satisfaction subscale demonstrated good internal consistency across waves, Cronbach's αs = .83–86.

#### MI

MI during military service was retrospectively assessed at W2 using the nine‐item Moral Injury Events Scale (MIES; Nash et al., 2013). The MIES measures exposure to PMIEs (e.g., “I acted in ways that violated my own moral code”) and reactions to those experiences (e.g., “I am troubled by having acted in ways that violated my moral code”). Items are scored on a 6‐point Likert‐type scale ranging from 1 (*strongly disagree*) to 6 (*strongly agree*), with higher values reflecting stronger endorsement of MI‐related experiences and reactions. Prior work has shown the MIES to have satisfactory internal reliability and construct validity in military and veteran samples (Bryan et al., 2016; Nash et al., 2013). Mean scores (possible range: 1–6) were calculated separately for MI from perpetration (MI‐Self; four items), MI from betrayal (MI‐Betray; three items), and MI from witnessing (MI‐Witness; two items). MI‐Self represents self‐directed MI, and MI‐Betray and MI‐Witness represent different dimensions of other‐directed MI. In the present sample, the three subscales demonstrated good‐to‐excellent internal consistency, MI‐Self: Cronbach's α = .94, MI‐Betray: α = .81; MI‐Witness: Spearman‐Brown *ρ* = .87.

#### PTSD symptoms

PTSD symptoms were assessed at W1 using the five‐item Primary Care PTSD screen (PC‐PTSD‐5; Prins et al., 2016). W1 PTSD data were used for the following reasons: (a) a survey administration error made W2 PTSD data unusable; (b) the PC‐PTSD‐5 was no longer administered starting at W3; and (c) the MIES was administered retrospectively, so W1 PTSD was considered the most comparable time point available. PC‐PTSD‐5 items reflect the *Diagnostic and Statistical Manual of Mental Disorders* (5th ed.; *DSM‐5*; American Psychiatric Association [APA], 2013) criteria for PTSD (e.g., “Have you had nightmares about the event(s) or thought about the event(s) when you did not want to?”). The measure focuses on symptom presence over the past month using binary response options (i.e., “yes” or “no”). The measure has been shown to have excellent diagnostic accuracy with veterans (Prins et al., 2016). A sum score (possible range: 0–5) was calculated, with higher values representing higher levels of symptom endorsement. In the present sample, internal consistency for the PC‐PTSD‐5 was good, Cronbach's α = .87.

#### Symptoms of anxiety and depression

Core symptoms of anxiety (e.g., “Feeling nervous, anxious, or on edge”) and depression (e.g., “little interest or pleasure in doing things”) were assessed at all waves using the four‐item Patient Health Questionnaire (PHQ‐4; Kroenke et al., 2009). For the present study, W1 anxiety and depression data were used for model parsimony. The PHQ‐4 focuses on symptom severity over the past 2 weeks, with items rated on a 4‐point Likert‐type scale ranging from 0 (*not at all*) to 3 (*nearly every day*). This measure has demonstrated evidence of satisfactory internal reliability and construct validity with respect to functional status, doctor visits, and symptom interference with usual activities (Kroenke et al., 2009). Separate sum scores (possible range: 0–6) were calculated for anxiety and depression, with higher sum scores indicating higher levels of symptom severity. Both the anxiety, *ρ* = .90, and depression, *ρ* = .89, subscales demonstrated good reliability in this sample.

#### Covariates

Five covariates, measured at W1, that can have relevant associations with parenting were included in the analyses (Gupta et al., 2023; Morgan et al., 2022). The covariates included parent gender (0 = mother, 1 = father), military pay grade (1 = E1–E4 to 6 = O4–O10), W1 marital status (0 = not first and only marriage [e.g., divorced] to 1 = first and only marriage), and number of deployments (*M* = 2.25, *SD* = 2.19). In addition, combat exposure was included as a covariate and assessed using the Deployment Risk and Resilience Inventory–2 Warfare Exposure–Short Form (DRRI‐2‐WF; Bovin et al., 2023). Items were scored on a scale of 0 (*never*) to 3 (*many times*), and sum scores were computed, with higher sum scores reflecting higher levels of exposure (*M* = 6.22*, SD* = 7.66; possible range: 0–27). In this sample, the DRRI‐2‐WF demonstrated excellent reliability, Cronbach's α = .93.

### Data analysis

Changes in positive parenting practices and satisfaction from W1 to W6 were examined using PP‐LGCM (Preacher et al., 2008) because this modeling approach allows for changes in multiple variables to be examined simultaneously. PP‐LGCMs have two major components: (a) the *intercept*, which represents the average starting value of the variables of interest, and (b) the *slope*, which captures average change over time in the variables of interest. Two series of individual LGCMs with no predictors or controls were estimated: one each for positive parenting practices and parenting satisfaction. Model 1 included random intercepts; Model 2 included random intercepts and random linear slopes; Model 3 included random intercepts, random linear slopes, and random quadratic slopes; and Model 4 included random intercepts and random latent basis slopes. To allow the models to be estimable and interpretable, the following elements were specified: (a) factor loadings for the intercept were set to 1.0, and the means of the observed indicators were constrained to 0; (b) factor loadings for linear slopes were set to 0, 0.5, 1.0, 1.5, 2.0, and 2.5; (c) factor loadings for quadratic slopes were set to the squared value of the linear slope loadings; and (d) factor loadings for the latent basis slopes were freely estimated for all waves except the first and last, which were fixed at 0 and 1.0, respectively. Estimating the latent basis model this way allows for the factor loadings calculated by the model to be interpreted as the percentage of the total average change, across all waves, that has occurred in the outcome by a given wave (McNeish, 2020). In addition, intercepts and slopes were allowed to covary, as were the residuals (i.e., unexplained error variance) for the observed indicators at corresponding time points.

All individual LGCMs were estimated with a robust maximum likelihood estimator (MLR), which corrects standard errors when data are nonnormal. The following common model fit indices and interpretation guidelines (Hu & Bentler, 1999) were used to assess model fit: scaled chi‐square, with lower values with nonsignificant *p* values preferred; robust comparative fit index (CFI), with values of .95 and above indicating good fit; robust root mean square error of approximation (RMSEA), with values of .06 or below representing good fit; and standardized root mean residual (SRMR), with values of .08 or below representing good fit. Because the estimated models were nested, scaled chi‐square difference tests were used to examine if lessening model restrictions resulted in statistically significant improvements in model fit. An additional model selection criterion was whether the variances of the intercept and slope were statistically significant, as a nonsignificant variance could signal that part of the model is unnecessary.

After selecting the best‐fitting models for both positive parenting practices and parenting satisfaction, a PP‐LGCM with predictor and control variables was specified using MLR and the same model fit guidelines as previously described. Missingness for the predictor and control variables ranged from 0% to 0.3%. Missingness for the positive parenting practices variable ranged from 0% to 0.2% across waves, and missingness for the parenting satisfaction variable ranged from 0% to 0.1% across waves. Full information maximum likelihood (FIML), which uses all available information in a dataset, was used to handle missing data in all of the specified growth models. All models were estimated using the *lavaan* package (Version 0.6‐19; Rosseel, 2012) in R (Version 4.5.2).

## RESULTS

### Descriptive statistics and correlations among variables

Table 2 contains descriptive statistics for and correlations among the primary study variables. All correlations were statistically significant, *p* < .001–*p* = .001, and in the expected direction (e.g., PTSD, anxiety, depression, and MI were negatively associated with positive parenting practices and satisfaction). Correlations among the covariates and the parenting variables were small across all waves (see Supplementary Table S1). On average, post‐9/11 veterans reported high levels of positive parenting practices (range: 4.49–4.61) and satisfaction (range: 4.42–4.56) and low levels of psychological distress (range: 1.11–1.41) and MI (range: 1.63–2.25).

**TABLE 2:** Primary study variable correlations and descriptive statistics

Variable	PTSD	Anx	Dep	MIS	MIB	MIW	W1 PP	W2 PP	W3 PP	W4 PP	W5 PP	W6 PP	W1 PS	W2 PS	W4 PS	W5 PS	W6 PS
PTSD	–	.66	.62	.30	.31	.31	−.24	−.23	−.22	−.21	−.22	−.22	−.21	−.17	−.21	−.23	−.22
Anx		–	.76	.31	.36	.34	−.26	−.28	−.22	−.23	−.24	−.18	−.24	−.21	−.25	−.22	−.18
Dep			–	.30	.35	.29	−.31	−.32	−.30	−.25	−.27	−.22	−.28	−.25	−.26	−.24	−.22
MIS				–	.50	.58	−.18	−.22	−.18	−.19	−.23	−.19	−.18	−.15	−.17	−.15	−.16
MIB					–	.61	−.20	−.26	−.17	−.19	−.20	−.17	−.19	−.18	−.20	−.17	−.17
MIW						–	−.17	−.20	−.15	−.14	−.17	−.16	−.15	−.15	−.14	−.12	−.12
W1 PP							–	.55	.52	.46	.48	.46	.66	.43	.43	.42	.37
W2 PP								–	.58	.54	.54	.50	.53	.64	.50	.48	.41
W3 PP									–	.60	.60	.59	.50	.48	.53	.53	.48
W4 PP										–	.61	.62	.46	.45	.69	.53	.50
W5 PP											–	.66	.47	.48	.54	.70	.55
W6 PP												–	.45	.41	.53	.56	.68
W1 PS													–	.61	.61	.55	.55
W2 PS														–	.62	.60	.54
W4 PS															–	.70	.68
W5 PS																–	.72
W6 PS																	–
*M*	1.41	1.60	1.11	1.65	2.17	2.56	4.56	4.61	4.57	4.55	4.56	4.49	4.54	4.56	4.50	4.50	4.42
*SD*	1.81	1.8	1.60	1.18	1.41	1.62	0.58	0.55	0.54	0.57	0.55	0.61	0.69	0.68	0.71	0.68	0.78

*Note*: All correlations were statistically significant at *p* < .001 – *p* = .001. Descriptive statistics and correlations were produced using robust maximum likelihood estimation with full information maximum likelihood to handle missing data (*n* = 1,078). PTSD = posttraumatic stress disorder; W = Wave; Anx = anxiety; dep = depression; MIS = moral injury–self; MIB = moral injury–betrayal; MIW = moral injury–witness; PP = parenting practices; PS = parenting satisfaction.

Table 3 provides fit statistics and the results of the scaled chi‐square difference tests for the individual LGCMs for positive parenting practices and satisfaction. For both parenting outcomes, Model 4 was retained as the best‐fitting, most parsimonious model. Though the scaled chi‐square difference test alone would have identified Model 3 as the best‐fitting model for positive parenting practices, the variance of the quadratic slope was not statistically significant, *p* = .082. For this reason, Model 4 was preferred. In reviewing the final models for positive parenting practices and satisfaction, it was observed that for both Model 3 and Model 4, the correlation between the intercept and slope was nonsignificant, and the estimated factor loading for the second wave was nonsignificant. Constraining the correlations, positive parenting practices: Δ*χ* ^2^(1) = 0.04, *p* = .838; parenting satisfaction: Δχ^2^(1) = 0.15, *p* = .699, and W2 factor loadings, positive parenting practices: Δχ^2^(1) = 1.32, *p* = .251; parenting satisfaction: Δχ^2^(1) = 0.96, *p* = .327, to zero did not significantly worsen model fit, so these parameters were constrained to zero in the PP‐LGCM.

**TABLE 3:** Individual latent growth curve model fit statistics and comparisons

Model	Standard χ^2^	Scaled χ^2^	*df*	Robust CFI	Robust RMSEA	90% CI	SRMR	Δχ^2^	*df*
Positive parenting practices									
M1. Random intercept	170.54^***^	104.32^***^	19	.95	.08	[.06, .10]	.06		
M2. Random intercept, random linear slope	44.30^***^	27.27^*^	16	.99	.03	[.01, .05]	.03	M1 vs. M2: 74.71^***^	3
M3. Random intercept, random linear slope, random quadratic slope	24.59^*^	15.04	12	1.0	.02	[.00, .05]	.02	M2 vs. M3: 12.38 (4)^*^	4
M4. Random intercept, random latent basis slope	24.05^*^	16.49	12	1.0	.02	[.00, .05]	.02	M2 vs. M4: 9.54	4
Parenting satisfaction									
M1. Random intercept	213.68^***^	129.78^***^	13	.93	.12	[.10, .14]	.08		
M2. Random Intercept, random linear slope	41.71^***^	27.37^**^	10	.99	.05	[.03, .07]	.03	M1 vs. M2: 83.67^***^	3
M3. Random intercept, random linear slope, random quadratic slope	26.07^***^	18.47^*^	6	.99	.05	[.03, .08]	.03	M2 vs. M3: 9.24	4
M4. Random intercept, random latent basis slope	24.72^**^	18.82^*^	7	1.0	.04	[.02, .07]	.02	M2 vs. M4: 8.43^*^	3

*Note*: The scaled chi‐square difference test uses the standard chi‐square value per model in its calculation, not the scaled chi‐square value. Model 4 was determined to be the best‐fitting, most parsimonious model for positive parenting practices and parenting satisfaction. M = Model; *df* = degrees of freedom; CFI = comparative fit index; RMSEA = root mean square error of approximation; CI = confidence interval; SRMR = standardized root mean residual.

* *p* < .05. ***p < .01. ***p < .001.

### Predictors of positive parenting practices and satisfaction

In the unconditional PP‐LGCM, scaled χ^2^(46) = 52.44, *p* = .239, robust CFI = 1.0, robust RMSEA = .01, 90% confidence interval (CI) [.00, .03], robust SRMR = .02, the slope means for positive parenting practices, α = −.09, *p* < .001, and satisfaction, α = −.13, *p* < .001, were both negative, which indicates declines in positive parenting practices and satisfaction over time. The factor loadings for positive parenting practices indicated that 28%, 50%, and 56% of the total average change in this outcome occurred by W3, W4, and W5, respectively. For parenting satisfaction, the factor loadings indicated that 46% and 60% of the total average change in the outcome occurred by W4 and W5, respectively. In addition, the variance estimates of the intercepts and slopes for positive parenting practices, ψ_intercept_ = .17, ψ_slope_ = .11, and satisfaction, ψ_intercept_ = .29, ψ_slope_ = .17, were statistically significant, *p*s < .001.

Figure 2 displays the standardized regression path estimates from the statistically significant predictor variables to the intercepts and slopes of the PP‐LGCM, scaled χ^2^(123) = 143.70, *p* = .098, robust CFI = 1.0, robust RMSEA = .01, 90% CI [.00, .02], robust SRMR = .02. Table 4 provides more detail on the regression parameter estimates of the model (see Supplementary Table S2 for model results pertaining to latent intercept and slope covariances, means, and variances). The correlation between the intercepts of positive parenting practices and parenting satisfaction was large, *r* = .81, *p* < .001. A similar pattern was observed for the correlation between the slopes of positive parenting practices and parenting satisfaction, *r* = .73, *p* < .001. MI‐Betray and depression were the only statistically significant predictors of both baseline positive parenting practices and satisfaction. The estimates were negative, meaning that higher MI‐Betray and depression scores were related to lower positive parenting practices and satisfaction scores obtained within the first 3 months of the military‐to‐civilian transition. MI‐Self was also found to have a negative effect on baseline positive parenting practices scores. With respect to rates of change, only PTSD scores were found to be predictive of change over time in positive parenting practices and satisfaction scores. More specifically, post‐9/11 veterans with higher PTSD scores within the first 3 months of the military‐to‐civilian transition experienced faster declines in positive parenting practices and satisfaction over the 2.5‐year study period compared to those with lower PTSD scores. To determine whether these findings were dependent on the set of control variables used, the analysis was rerun without covariates. The findings were highly similar to the model with the covariates (see Supplementary Table S3). Sensitivity analyses were also conducted to determine if the decision to constrain the W2 factor loadings for the parenting constructs to zero impacted the findings. The results of these analyses were highly similar to those from the original analysis (see Supplementary Tables S4 and S5).

**FIGURE 2:**
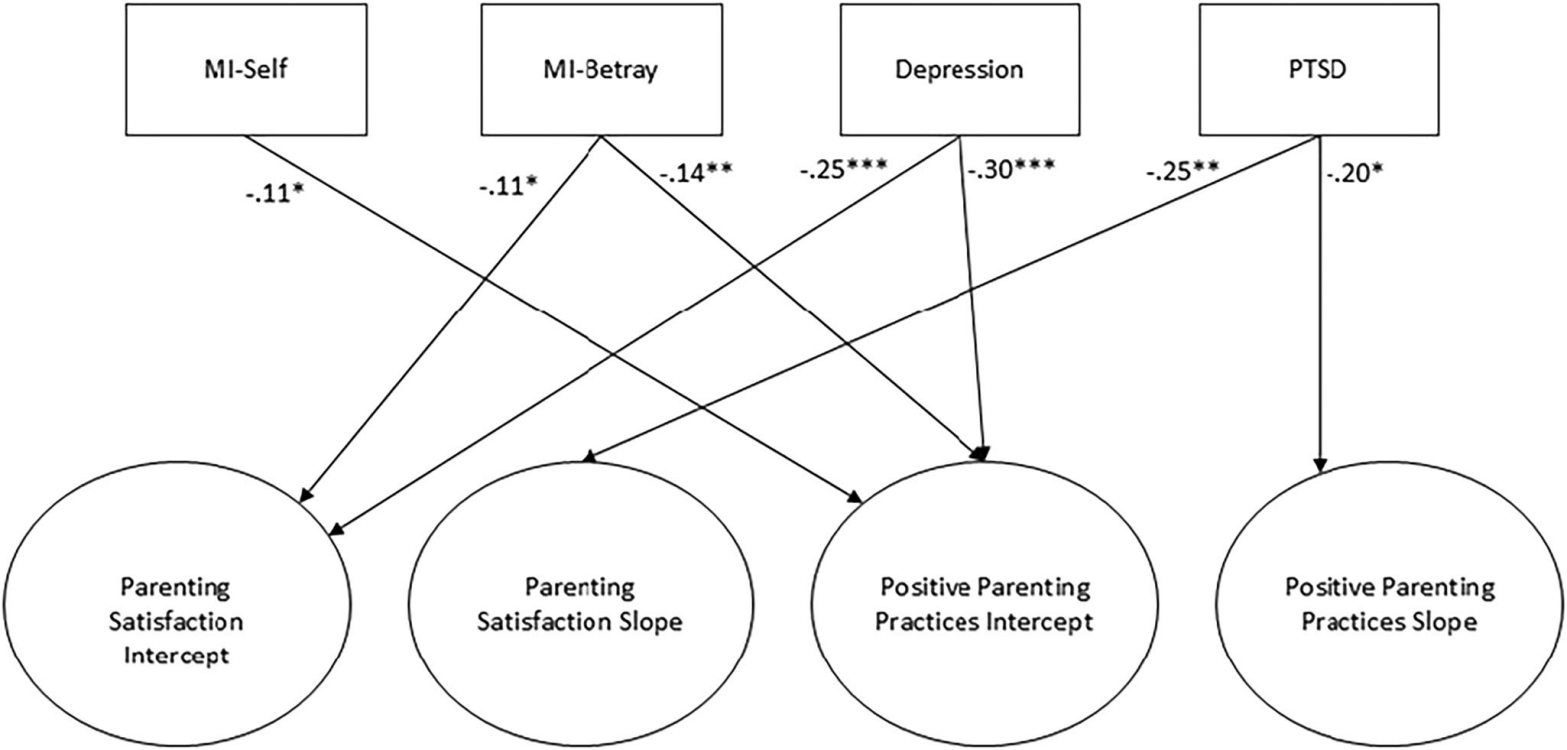
Effects of statistically significant predictors on positive parenting practices and parenting satisfaction *Note*: Observed variables, residual variances, residual covariances, nonsignificant predictors, nonsignificant paths, and control variables are not shown for presentational clarity. Standardized regression coefficients are shown. MI = moral injury; PTSD = posttraumatic stress disorder. *p < .05. **p < .01. ***p < .001.

**TABLE 4:** Results of the parallel process latent growth curve modeling with predictor and control variables

Regression parameter	*B*	*SE*	95% CI	*p*	β
Positive parenting practices intercept					
MI‐self	−0.04	0.02	[−0.07, −0.002]	.036	−.11
MI‐witness	0.004	0.01	[−0.02, 0.03]	.752	.02
MI‐betray	−0.04	0.01	[−0.07, −0.01]	.004	−.14
Depression	−0.08	0.02	[−0.11, −0.05]	< .001	−.30
Anxiety	−0.01	0.01	[−0.04, 0.01]	.258	−.07
PTSD	0.004	0.01	[−0.02, 0.02]	.728	.02
Gender^a^	−0.11	0.04	[−0.18, −0.04]	.003	−.09
Marital status^b^	−0.03	0.03	[−0.09, 0.02]	.254	−.04
Combat exposure	−0.003	0.002	[−0.01, 0.002]	.233	−.05
Number of deployments	−0.004	0.01	[−0.02, 0.01]	.520	−.02
Pay grade	−0.02	0.01	[−0.03, <0.001]	.055	−.06
Positive parenting practices slope					
MI‐self	−0.02	0.02	[−0.06, 0.02]	.332	−.07
MI‐witness	−0.01	0.02	[−0.04, 0.02]	.658	−.03
MI‐betray	0.02	0.02	[−0.01, 0.05]	.127	.10
Depression	0.03	0.02	[−0.01, 0.07]	.143	.14
Anxiety	0.02	0.02	[−0.01, 0.06]	.145	.14
PTSD	−0.04	0.01	[−0.06, −0.01]	.010	−.20
Gender^a^	−0.03	0.05	[−0.12, 0.06]	.557	−.03
Marital status^b^	0.03	0.04	[−0.04, 0.11]	.373	.05
Combat exposure	0.003	0.003	[−.002, 0.01]	.269	.07
Number of deployments	−0.01	0.01	[−0.02, 0.01]	.383	−.05
Pay grade	0.01	0.01	[−0.01, 0.03]	.217	.06
Parenting satisfaction intercept					
MI‐self	−0.04	0.02	[−0.08, 0.01]	.085	−.08
MI‐witness	0.004	0.02	[−0.03, 0.03]	.810	.01
MI‐betray	−0.04	0.02	[−0.08, −0.01]	.026	−.11
Depression	−0.08	0.02	[−0.13, −0.04]	< .001	−.25
Anxiety	−0.02	0.02	[−0.05, 0.01]	.207	−.07
PTSD	0.01	0.02	[−0.02, 0.04]	.459	.04
Gender^a^	−0.09	0.05	[−0.19, 0.003]	.059	−.06
Marital status^b^	0.04	0.04	[−0.04, 0.12]	.290	.04
Combat exposure	−0.004	0.003	[−0.01, 0.001]	.137	−.06
Number of deployments	−0.01	0.01	[−0.02, 0.01]	.340	−.03
Pay grade	−0.03	0.01	[−0.05, −0.01]	.002	−.10
Parenting satisfaction slope					
MI‐self	−0.02	0.03	[−0.07, 0.03]	.504	−.05
MI‐witness	0.02	0.02	[−0.01, 0.05]	.121	.09
MI‐betray	−0.01	0.02	[−0.04, 0.02]	.583	−.03
Depression	0.03	0.02	[−0.02, 0.07]	.228	.10
Anxiety	0.02	0.02	[−0.02, 0.06]	.272	.10
PTSD	−0.06	0.02	[−0.09, −0.02]	.001	−.25
Gender^a^	−0.05	0.06	[−0.16, 0.07]	.436	−.04
Marital status^b^	0.08	0.05	[−0.01, 0.17]	.075	.09
Combat exposure	0.003	0.003	[−0.003, 0.01]	.277	.06
Number of deployments	−0.01	0.01	[−0.03, 0.003]	.111	−.08
Pay grade	−0.002	0.01	[−0.03, 0.02]	.888	−.01

*Note*. CI = confidence interval; MI = moral injury; PTSD = posttraumatic stress disorder. ^a^Gender: 0 = mother, 1 = father.

^b^ Marital status: 0 = not first and only marriage, 1 = first and only marriage.

## DISCUSSION

This study sought to examine the effects of psychological symptoms on post‐9/11 veterans’ positive parenting practices and satisfaction by emphasizing the role of three types of MI (i.e., MI related to one's actions or inactions, betrayal‐related MI, and MI one has witnessed). On average, post‐9/11 veterans’ reports of positive parenting practices and satisfaction declined over time. This finding aligns with previous work demonstrating that parenting behaviors and attitudes (e.g., warmth, involvement, satisfaction) tend to decrease across time in civilian (Nomaguchi & Milkie, 2020) and military‐connected families (Flittner O'Grady et al., 2018). The results of the conditional PP‐LGCM also revealed strong correlations between the baseline values of positive parenting practices and parenting satisfaction, *r* = .81, and the slopes of these parenting constructs, *r* = .73. These correlations indicate that veteran parents who reported higher levels of positive parenting practices at baseline also tended to report higher baseline levels of parenting satisfaction. Similarly, veteran parents who experienced steeper declines in positive parenting practices over time also tended to experience steeper declines in parenting satisfaction over time. These correlational findings correspond to the initial validation work performed on the Well‐Being Inventory subscales used to measure positive parenting practices and parenting satisfaction (Vogt et al., 2019) and further support the concept that these parenting constructs operate in tandem for veteran parents.

MI related to acts of betrayal and depressive symptoms were the only psychological predictors of baseline positive parenting practices and satisfaction. The effects indicated that higher MI‐Betrayal scores and more severe depressive symptoms were associated with lower levels of positive parenting practices and satisfaction during the early transition to civilian life. MI‐Self scores (i.e., MI related to one's actions or inaction) were also found to negatively predict baseline positive parenting practices. In general, the findings for MI align with the cross‐sectional results reported by Ne'eman‐Haviv et al. (2025); however, their study did not find a direct association between betrayal‐based MI and parenting. This discrepancy could be due to differences in measurement, study design, veteran sample, or a combination of these factors. Similarly, the findings for depression are in line with prior cross‐sectional studies (Lovejoy et al., 2000; Ryan et al., 2017). Regarding changes in positive parenting practices and satisfaction over time, anxiety, depression, or any type of MI were not found to be statistically significant predictors. Post‐9/11 veterans with higher PTSD scores at baseline, however, evidenced more rapid declines in positive parenting practices and satisfaction over the study period. These findings confirm the sustained negative impact of PTSD symptoms on military and veteran parenting found in prior studies (Kizer & Le Menestrel, 2019) and potentially indicate that, although C‐BIT can be extended to include mental health conditions beyond PTSD, symptoms of PTSD may be the most salient in terms of continued disruptions in parenting cognitions, attitudes, and behaviors.

Collectively, the findings indicate that MI might have a small and short‐term impact on parenting behaviors and satisfaction_._ The results of this study also suggest that MI from betrayal, compared to MI from self‐directed actions or inactions or witnessing PMIEs, may be more problematic for parents in the early part of the military‐to‐civilian transition, as this was the only type of MI that was related to both parenting outcomes. However, the measure of MI was a measure of the occurrence of PMIE exposure and the corresponding feeling of being troubled or betrayed by these events, and it was not a measure of specific psychological symptoms, as is the case with established measures of PTSD, depressive, and anxiety symptoms. It is possible that some of the relationally oriented psychological symptoms associated with MI are similar to, or partially overlap with, symptoms of depression, anxiety, or PTSD. Indeed, betrayal erodes trust, and previous research has revealed that feelings of betrayal are associated with the *DSM‐5* PTSD symptom clusters of avoidance and negative alterations in cognitions and mood (APA, 2013; Levi‐Belz et al., 2020). Moreover, these PTSD symptom clusters have been found to negatively impact parenting and the parent–child relationship (Lauterbach et al., 2007; Sherman et al., 2016; Wamser‐Nanney & Sager, 2022). Thus, PTSD symptom clusters may occupy a mediating role between betrayal‐related MI and parenting outcomes. This finding would be consistent with the connections found between PTSD symptoms and parenting behavior in the C‐BIT model, as well as with the study's finding that experiencing PTSD predicted change over time in parenting outcomes, whereas MI did not. Betrayal‐related MI could activate any of the three processes implicated in the C‐BIT model (i.e., behavioral avoidance and accommodation, cognitive processes and thematic content, and emotional disturbances), which, in turn, could compound PTSD symptomology and impair parenting behaviors and satisfaction. This is an area for future research to explore, and, given the C‐BIT model's general applicability to mental health concerns, this research should also consider depressive and anxious symptomatology.

The findings of this study should be interpreted with several limitations in mind. First, all data were gathered using self‐report measures, which means the data are susceptible to multiple forms of bias. Second, the MIES was employed retrospectively; in addition, the MIES has multiple limitations, such as generic item wording and simultaneous assessment of experiences and reactions. The simultaneous measurement of MI‐related experiences and reactions, combined with the singular assessment of MI, prevented us from being able to explore possible mediation pathways from MI subtypes to parenting outcomes via PTSD, depressive, or anxiety symptoms. Third, FIML was employed to account for missing data. This technique works well when data are missing randomly, but the missing at random assumption cannot be proven. If the data are not random, the model parameter estimates will be biased. Fourth, MI, PTSD, depression, and anxiety were treated as time‐invariant predictors because of data limitations and the prioritization of model parsimony. These variables are likely to change across time, and future research that includes data that will allow this variability to be appropriately modeled is needed. Finally, the sampling frame for the TVMI study was post‐9/11 veterans who had transitioned out of the military between August 2016 and November 2016. Thus, the extent to which the post‐9/11 veteran parents in this study represent all U.S. veteran parents is unclear.

Despite these limitations, the current study contributes to the MI and parenting literature by being among the first to investigate how MI relates to post‐9/11 veterans’ parenting across time within the context of other relevant mental health, demographic, and military‐specific variables. MI research focused on the family system—and parenting more specifically—is in need of increased attention. Overall, the results of the present study indicate that betrayal‐related MI; depressive symptoms; and, to a lesser extent, MI stemming from one's own actions or inactions, may be important factors influencing post‐9/11 veteran parents’ parenting behaviors and satisfaction within the first 3 months of the military‐to‐civilian transition. The results also suggest that PTSD can have a persistent, negative effect on parenting behaviors and satisfaction. Thus, although PTSD, depression, and MI operate in different capacities, all are likely important to consider when trying to understand the nuances and complexities of parenting within the U.S. veteran population. Continued research is needed to help researchers, practitioners, and clinicians be able to provide the best support possible to service members, veterans, and their families.

## AUTHOR NOTE

The Veterans Metrics Initiative (TVMI) research was managed by the Henry M. Jackson Foundation for the Advancement of Military Medicine, Inc. (HJF), and it was collaboratively sponsored by the Bob Woodruff Foundation, Health Net Federal Services, HJF, Lockheed Martin Corporation, Marge and Philip Odeen, May and Stanley Smith Charitable Trust, National Endowment for the Humanities, Northrop Grumman, Prudential, Robert R. McCormick Foundation, Rumsfeld Foundation, Schultz Family Foundation, Heinz Endowments, U.S. Department of Veterans Affairs (VA) Health Services Research and Development Service, Walmart Foundation, and Wounded Warrior Project. The Clearinghouse for Military Family Readiness at Penn State is the result of a partnership funded by the U.S. Department of Defense (DoD) between the Office of the Deputy Assistant Secretary of Defense for Military Community and Family Policy and the U.S. Department of Agriculture (USDA)’s National Institute of Food and Agriculture through a cooperative agreement with the Pennsylvania State University. This work leverages funds from the USDA's National Institute of Food and Agriculture and Hatch Appropriations.

The authors wish to thank the many individuals whose support through the different stages of the research effort was instrumental to this publication. In particular, the authors thank the funders, whose financial support allowed us to execute this project, and the participants, without whose valuable perspective we could not have conducted the analyses to test the research questions herein.

## OPEN PRACTICES STATEMENT

The study reported in this article was not formally preregistered. De‐identified data have been made publicly available at the Inter‐University Consortium for Political and Social Research and can be accessed at https://www.icpsr.umich.edu/web/ICPSR/studies/38051/summary.

## AUTHOR CONTRIBUTIONS

**Ryan P. Chesnut**: conceptualization, methodology, formal analysis, writing—original draft, writing—review and editing, data curation, visualization. **Jennifer K. Karre**: conceptualization, methodology, writing—original draft, writing—review and editing. **Cameron B. Richardson**: conceptualization, methodology, writing—original draft, writing—review and editing. **Keith R. Aronson**: writing—review and editing, supervision. **Daniel F. Perkins**: funding acquisition, supervision, resources, writing—review and editing.

## Supporting information



Supporting Information
