# Supplementary material for: Veterans’ positive parenting practices and satisfaction over time: Examining the impact of moral injury, posttraumatic stress, and internalizing symptoms
Source: J Trauma Stress. 2026 Jun 1;39(5):857–70. doi: 10.1002/jts.70083 (PMC13639212; doi:10.1002/jts.70083)
Supplement: Supplementary file 1 [file JTS-39-857-s001.docx]

**Supplemental Table 1**

*Covariate Correlations with Parenting Variables across Waves*

|  | W1 PP | W2 PP | W3 PP | W4 PP | W5 PP | W6 PP | W1 PS | W2 PS | W4 PS | W5 PS | W6 PS |
| --- | --- | --- | --- | --- | --- | --- | --- | --- | --- | --- | --- |
| Gender | -0.11*** | -0.06 | -0.11*** | -0.08** | -0.07* | -0.09** | -0.08* | -0.06* | -0.04 | -0.05 | -0.08** |
| Marital Status | -0.01 | -0.02 | 0.003 | -0.01 | -0.002 | 0.01 | 0.05 | 0.03 | 0.07* | 0.06 | 0.08* |
| Combat Exposure | -0.15*** | -0.14*** | -0.15*** | -0.11** | -0.10** | -0.13*** | -0.17*** | -0.11** | -0.14*** | -0.13*** | -0.15*** |
| Deployment No. | -0.07* | -0.02 | -0.06* | -0.05 | -0.08** | -0.05 | -0.08** | -0.06* | -0.07* | -0.10** | -0.11*** |
| Paygrade | 0.01 | -0.001 | 0.05 | 0.02 | 0.04 | 0.02 | -0.03 | -0.03 | -0.03 | -0.03 | -0.04 |

*Note.* No. = Number. *p < .05; **p < .01; ***p < .001. PP = Parenting Practices; PS = Parenting Satisfaction.

**Supplemental Table 2**

*PP-LGCM Intercept and Slope Covariance, Mean, and Variance Parameter Estimates*

| *Intercept and Slope Covariance Parameters* | COV | *SE* | *p* | 95% CI | *r* |
| --- | --- | --- | --- | --- | --- |
| Positive Parenting Practices Intercept with Parenting Satisfaction Intercept | 0.14 | 0.01 | <0.001 | [0.11, 0.17] | 0.81 |
| Positive Parenting Practices Slope with  Parenting Satisfaction Slope | 0.10 | 0.02 | <0.001 | [0.06, 0.13] | 0.73 |
| *Intercept and Slope Means* | *α* | *SE* | *p* | 95% CI | CSS *α* |
| Positive Parenting Practices Intercept | 4.71 | 0.03 | <0.001 | [4.64, 4.77] | 11.46 |
| Parenting Satisfaction Intercept | 4.61 | 0.05 | <0.001 | [4.51, 4.70] | 8.61 |
| Positive Parenting Practices Slope | -0.09 | 0.04 | 0.02 | [-0.17, -0.01] | -0.28 |
| Parenting Satisfaction Slope | -0.15 | 0.06 | 0.01 | [-0.26, -0.04] | -0.35 |
| *Intercept and Slope Variance* | *ψ* | *SE* | *p* | 95% CI | CSS *ψ* |
| Positive Parenting Practices Intercept | 0.13 | 0.01 | <0.001 | [0.11, 0.15] | 0.77 |
| Parenting Satisfaction Intercept | 0.24 | 0.02 | <0.001 | [0.20, 0.28] | 0.84 |
| Positive Parenting Practices Slope | 0.10 | 0.02 | <0.001 | [0.06, 0.15] | 0.95 |
| Parenting Satisfaction Slope | 0.16 | 0.03 | <0.001 | [0.11, 0.22] | 0.95 |

*Note.* COV = Covariance; α = Latent Variable Mean; ψ = Latent Variable Variance; CSS = Completely Standardized Solution.

**Supplemental Table 3**

*PP-LGCM Results Excluding Control Variables*

| *Regression Parameters* | *B* | *SE* | *p* | 95% CI | *β* |
| --- | --- | --- | --- | --- | --- |
| Positive Parenting Practices Intercept |  |  |  |  |  |
| MI-Self | -0.04 | 0.02 | 0.04 | [-0.07, -0.001] | -0.10 |
| MI-Witness | 0.003 | 0.01 | 0.81 | [-0.02, 0.03] | 0.01 |
| MI-Betray | -0.04 | 0.01 | 0.01 | [-0.07, -0.01] | -0.13 |
| Depression | -0.08 | 0.02 | <0.001 | [-0.11, -0.05] | -0.31 |
| Anxiety | -0.01 | 0.01 | 0.52 | [-0.03, 0.02] | -0.04 |
| PTSD | -0.004 | 0.01 | 0.68 | [-0.02, 0.02] | -0.02 |
| Positive Parenting Practices Slope |  |  |  |  |  |
| MI-Self | -0.02 | 0.02 | 0.35 | [-0.06, 0.02] | -0.07 |
| MI-Witness | -0.01 | 0.02 | 0.75 | [-0.04, 0.03] | -0.02 |
| MI-Betray | 0.02 | 0.02 | 0.19 | [-0.01, 0.05] | 0.08 |
| Depression | 0.03 | 0.02 | 0.14 | [-0.01, 0.07] | 0.14 |
| Anxiety | 0.02 | 0.02 | 0.19 | [-0.01, 0.05] | 0.12 |
| PTSD | -0.03 | 0.01 | 0.01 | [-0.06, -0.01] | -0.18 |
| Parenting Satisfaction Intercept |  |  |  |  |  |
| MI-Self | -0.03 | 0.02 | 0.14 | [-0.08, 0.01] | -0.07 |
| MI-Witness | 0.001 | 0.02 | 0.96 | [-0.03, 0.03] | 0.002 |
| MI-Betray | -0.04 | 0.02 | 0.04 | [-0.78, -0.001] | -0.10 |
| Depression | -0.08 | 0.02 | <0.001 | [-0.12, -0.04] | -0.24 |
| Anxiety | -0.01 | 0.02 | 0.41 | [-0.05, 0.02] | -0.05 |
| PTSD | -0.002 | 0.01 | 0.86 | [-0.03, 0.03] | -0.01 |
| Parenting Satisfaction Slope |  |  |  |  |  |
| MI-Self | -0.01 | 0.03 | 0.60 | [-0.06, 0.04] | -0.04 |
| MI-Witness | 0.03 | 0.02 | 0.09 | [-0.004, 0.06] | 0.10 |
| MI-Betray | -0.01 | 0.02 | 0.45 | [-0.05, 0.02] | -0.04 |
| Depression | 0.03 | 0.02 | 0.20 | [-0.01, 0.07] | 0.11 |
| Anxiety | 0.02 | 0.02 | 0.31 | [-0.02, 0.06] | 0.09 |
| PTSD | -0.05 | 0.02 | 0.001 | [-0.09, -0.02] | -0.24 |

*Note.* B = Unstandardized Regression Coefficient; SE = Standard Error; *p* = p-value; CI = Confidence Interval; *β =* Standardized Regression Coefficient; MI = Moral Injury; PTSD = Post-traumatic Stress Disorder.

**Supplemental Table 4**

*Conditional PP-LGCM Results with Wave 2 Factor Loadings Freely Estimated*

| *Regression Parameters* | *B* | *SE* | *p* | 95% CI | *β* |
| --- | --- | --- | --- | --- | --- |
| Positive Parenting Practices Intercept |  |  |  |  |  |
| MI-Self | -0.04 | 0.02 | 0.03 | [-0.07, -0.004] | -0.11 |
| MI-Witness | 0.003 | 0.01 | 0.77 | [-0.02, 0.03] | 0.01 |
| MI-Betray | -0.04 | 0.01 | 0.003 | [-0.07, -0.01] | -0.14 |
| Depression | -0.08 | 0.02 | <0.001 | [-0.11, -0.04] | -0.29 |
| Anxiety | -0.01 | 0.01 | 0.28 | [-0.04, 0.01] | -0.06 |
| PTSD | 0.002 | 0.01 | 0.85 | [-0.02, 0.02] | 0.01 |
| Gender | -0.11 | 0.04 | 0.003 | [-0.18, -0.04] | -0.09 |
| Marital Status | -0.03 | 0.03 | 0.27 | [-0.09, 0.03] | -0.04 |
| Combat Exposure | -0.003 | 0.002 | 0.21 | [-0.01, 0.002] | -0.05 |
| Number of Deployments | -0.003 | 0.01 | 0.58 | [-0.02, 0.01] | -0.02 |
| Paygrade | -0.02 | 0.01 | 0.05 | [-0.03, <.001] | -0.06 |
| Positive Parenting Practices Slope |  |  |  |  |  |
| MI-Self | -0.02 | 0.02 | 0.33 | [-0.06, 0.02] | -0.07 |
| MI-Witness | -0.01 | 0.01 | 0.65 | [-0.03, 0.02] | -0.03 |
| MI-Betray | 0.02 | 0.01 | 0.08 | [-0.003, 0.05] | 0.11 |
| Depression | 0.03 | 0.02 | 0.16 | [-0.01, 0.06] | 0.14 |
| Anxiety | 0.02 | 0.02 | 0.13 | [-0.01, 0.05] | 0.14 |
| PTSD | -0.03 | 0.01 | 0.01 | [-0.06, -0.01] | -0.20 |
| Gender | -0.03 | 0.04 | 0.46 | [-0.12, 0.05] | -0.04 |
| Marital Status | 0.03 | 0.03 | 0.36 | [-0.04, 0.10] | 0.05 |
| Combat Exposure | 0.003 | 0.003 | 0.20 | [-.002, 0.01] | 0.08 |
| Number of Deployments | -0.01 | 0.01 | 0.25 | [-0.03, 0.01] | -0.06 |
| Paygrade | 0.01 | 0.01 | 0.15 | [-0.004, 0.03] | 0.07 |
| Parenting Satisfaction Intercept |  |  |  |  |  |
| MI-Self | -0.04 | 0.02 | 0.08 | [-0.08, 0.01] | -0.09 |
| MI-Witness | 0.004 | 0.02 | 0.81 | [-0.03, 0.03] | 0.01 |
| MI-Betray | -0.04 | 0.02 | 0.03 | [-0.08, -0.01] | -0.11 |
| Depression | -0.08 | 0.02 | <0.001 | [-0.13, -0.04] | -0.25 |
| Anxiety | -0.02 | 0.02 | 0.21 | [-0.05, 0.01] | -0.07 |
| PTSD | 0.01 | 0.02 | 0.47 | [-0.02, 0.04] | 0.04 |
| Gender | -0.09 | 0.05 | 0.06 | [-0.18, 0.01] | -0.06 |
| Marital Status | 0.04 | 0.04 | 0.29 | [-0.04, 0.12] | 0.04 |
| Combat Exposure | -0.005 | 0.003 | 0.13 | [-0.01, 0.001] | -0.07 |
| Number of Deployments | -0.01 | 0.01 | 0.38 | [-0.02, 0.01] | -0.03 |
| Paygrade | -0.03 | 0.01 | 0.002 | [-0.05, -0.01] | -0.10 |
| Parenting Satisfaction Slope |  |  |  |  |  |
| MI-Self | -0.02 | 0.03 | 0.51 | [-0.07, 0.03] | -0.05 |
| MI-Witness | 0.02 | 0.02 | 0.12 | [-0.01, 0.05] | 0.10 |
| MI-Betray | -0.01 | 0.02 | 0.63 | [-0.04, 0.03] | -0.03 |
| Depression | 0.03 | 0.02 | 0.24 | [-0.02, 0.07] | 0.10 |
| Anxiety | 0.02 | 0.02 | 0.26 | [-0.02, 0.06] | 0.10 |
| PTSD | -0.06 | 0.02 | 0.001 | [-0.09, -0.02] | -0.25 |
| Gender | -0.05 | 0.06 | 0.40 | [-0.17, 0.07] | -0.04 |
| Marital Status | 0.08 | 0.05 | 0.07 | [-0.01, 0.17] | 0.09 |
| Combat Exposure | 0.004 | 0.003 | 0.27 | [-0.003, 0.01] | 0.07 |
| Number of Deployments | -0.02 | 0.01 | 0.09 | [-0.03, 0.002] | -0.08 |
| Paygrade | -0.001 | 0.01 | 0.94 | [-0.02, 0.02] | -0.004 |

*Note.* B = Unstandardized Regression Coefficient; SE = Standard Error; *p* = p-value; CI = Confidence Interval; *β =* Standardized Regression Coefficient; MI = Moral Injury; PTSD = Post-traumatic Stress Disorder; Gender (0=Mother; 1=Father); Marital Status (0=Not First and Only Marriage; 1=First and Only Marriage).

**Supplemental Table 5**

*Conditional PP-LGCM Results with Contral Variables Excluded and Wave 2 Factor Loadings Freely Estimated*

| *Regression Parameters* | *B* | *SE* | *p* | 95% CI | *β* |
| --- | --- | --- | --- | --- | --- |
| Positive Parenting Practices Intercept |  |  |  |  |  |
| MI-Self | -0.04 | 0.02 | 0.04 | [-0.07, -0.002] | -0.10 |
| MI-Witness | 0.003 | 0.01 | 0.83 | [-0.03, 0.01] | 0.01 |
| MI-Betray | -0.04 | 0.01 | 0.01 | [-0.07, -0.01] | -0.13 |
| Depression | -0.08 | 0.02 | <0.001 | [-0.11, -0.04] | -0.30 |
| Anxiety | -0.01 | 0.01 | 0.55 | [-0.03, 0.02] | -0.03 |
| PTSD | -0.006 | 0.01 | 0.58 | [-0.03, 0.01] | -0.02 |
| Positive Parenting Practices Slope |  |  |  |  |  |
| MI-Self | -0.02 | 0.02 | 0.36 | [-0.06, 0.02] | -0.07 |
| MI-Witness | -0.004 | 0.01 | 0.77 | [-0.03, 0.02] | -0.02 |
| MI-Betray | 0.02 | 0.01 | 0.14 | [-0.01, 0.05] | 0.09 |
| Depression | 0.03 | 0.02 | 0.16 | [-0.01, 0.06] | 0.14 |
| Anxiety | 0.02 | 0.02 | 0.18 | [-0.01, 0.05] | 0.12 |
| PTSD | -0.03 | 0.01 | 0.01 | [-0.06, -0.01] | -0.18 |
| Parenting Satisfaction Intercept |  |  |  |  |  |
| MI-Self | -0.03 | 0.02 | 0.14 | [-0.08, 0.01] | -0.07 |
| MI-Witness | 0.001 | 0.02 | 0.86 | [-0.03, 0.03] | 0.002 |
| MI-Betray | -0.04 | 0.02 | 0.04 | [-0.07, -0.001] | -0.10 |
| Depression | -0.08 | 0.02 | <0.001 | [-0.12, -0.04] | -0.24 |
| Anxiety | -0.01 | 0.02 | 0.41 | [-0.05, 0.02] | -0.05 |
| PTSD | -0.002 | 0.01 | 0.87 | [-0.03, 0.03] | -0.01 |
| Parenting Satisfaction Slope |  |  |  |  |  |
| MI-Self | -0.01 | 0.03 | 0.560 | [-0.06, 0.04] | -0.04 |
| MI-Witness | 0.03 | 0.02 | 0.109 | [-0.004, 0.06] | 0.10 |
| MI-Betray | -0.01 | 0.02 | 0.48 | [-0.05, 0.02] | -0.04 |
| Depression | 0.03 | 0.02 | 0.21 | [-0.02, 0.07] | 0.11 |
| Anxiety | 0.02 | 0.02 | 0.30 | [-0.02, 0.06] | 0.09 |
| PTSD | -0.06 | 0.02 | 0.001 | [-0.09, -0.02] | -0.24 |

*Note.* B = Unstandardized Regression Coefficient; SE = Standard Error; *p* = p-value; CI = Confidence Interval; *β =* Standardized Regression Coefficient; MI = Moral Injury; PTSD = Post-traumatic Stress Disorder.
